# Supplementary material for: Genomics Analysis Reveals the Potential Biocontrol Mechanism of Pseudomonas aeruginosa QY43 against Fusarium pseudograminearum
Source: J Fungi (Basel). 2024 Apr 21;10(4):298. doi: 10.3390/jof10040298 (PMC11050789; doi:10.3390/jof10040298)
Supplement: Supplementary file 1 [file jof-10-00298-s001.zip › jof-2956202-supplementary.pdf]

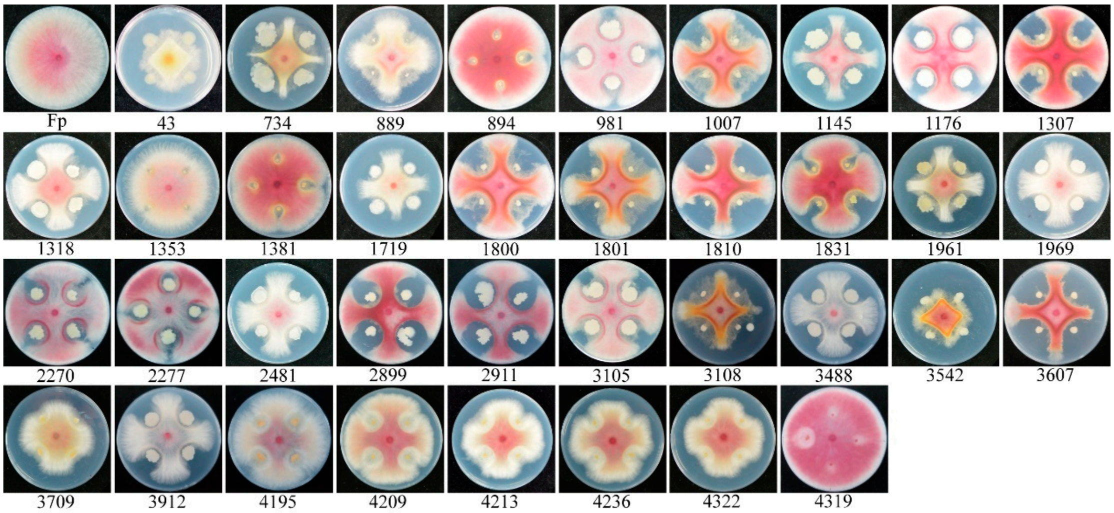

Figure S1. Confrontation culture of the 37 isolated strains and *Fusarium pseudograminearum* WZ-8A (Fp) on potato dextrose agar (PDA) plates. Colony morphology of WZ-8A on a PDA plate with or without biocontrol bacteria.

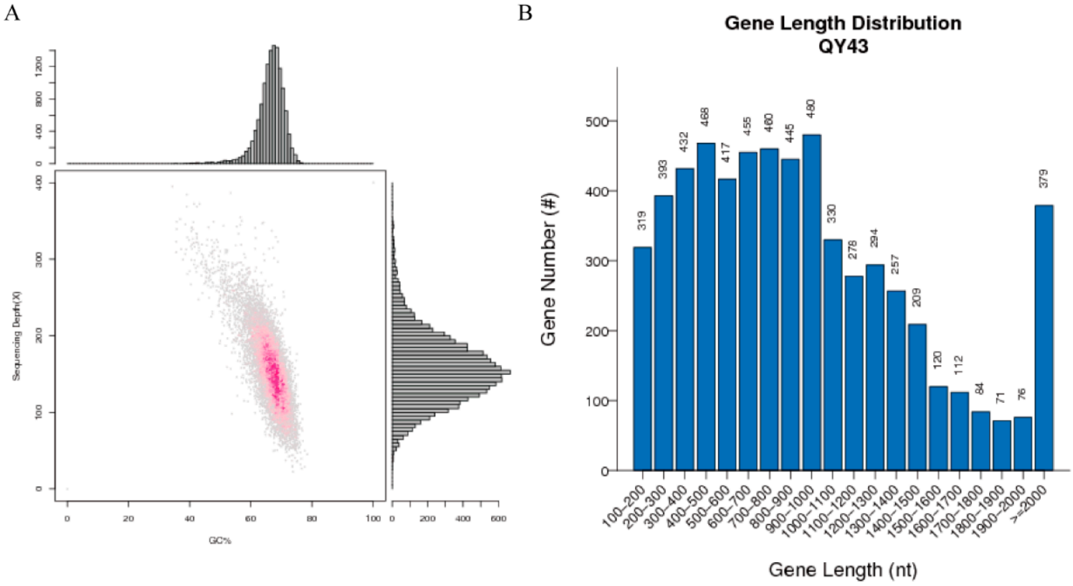

Figure S2. QY43 genomics including GC-depth analysis and gene length distribution. (A): GC content and depth correlative analysis. The GC content and average depth were calculated with 500 bp as the window without repetition, and the GC bias of the sequencing data was analyzed based on these results; (B): Gene length distribution.

Table S1. List of genes related to antagonistic activity and plant growth promotion (PGP) attributes from the isolated *P. aeruginosa* QY43 genome.

Biological control mechanism	Gene Name	Protein Names
antagonistic activities		
Phenazine-1-carboxamide acid PCA	QY43GL000735	(*phzG1*) probable pyrodoxamine 5'-phosphate oxidase
	QY43GL000736	(*phzF1*) putative phenazine biosynthesis protein
	QY43GL000737	(*phzE1*) phenazine biosynthesis protein PhzE
	QY43GL000738	(*phzD1*) phenazine biosynthesis protein PhzD, isochorismatase
	QY43GL000739	(*phzC1*) phenazine biosynthesis protein PhzC
	QY43GL000740	(*phzB1*) probable phenazine biosynthesis protein
	QY43GL000741	(*phzA1*) phenazine biosynthesis protein PhzA
	QY43GL002160	(*phzC1*) phenazine biosynthesis protein PhzC
	QY43GL003291	(*phzG1*) probable pyrodoxamine 5'-phosphate oxidase
	QY43GL003292	(*phzF1*) phenazine biosynthesis protein PhzF, isomerase
	QY43GL003293	(*phzE1*) phenazine biosynthesis protein PhzE
	QY43GL003294	(*phzC1*) phenazine biosynthesis protein PhzC
	QY43GL003295	(*phzD1*) phenazine biosynthesis protein PhzD, isochorismatase
	QY43GL003296	(*phzB2*) probable phenazine biosynthesis protein
	QY43GL003297	(*phzA2*) probable phenazine biosynthesis protein
Pyocyanin PYO	QY43GL000742	(*phzM*) phenazine-specific methyltransferase PhzM
	QY43GL000734	(*phzS*) flavin-containing monooxygenase
Phenazine-1-carboxamide PCN	QY43GL000054	(*phzH*) potential phenazine-modifying enzyme
	QY43GL003102	(*phzH*) potential phenazine-modifying enzyme
1-Hydroxyphenazine	QY43GL000734	(*phzS*) flavin-containing monooxygenase
Rhamnolipid	QY43GL004214	(*rhlC*) rhamnosyltransferase 2
	QY43GL004646	(*rhlA*) rhamnosyltransferase chain A
	QY43GL001502	(*rhlA*) rhamnosyltransferase chain A
	QY43GL001503	(*rhlB*) rhamnosyltransferase chain B
	QY43GL001504	(*rhlR*) transcriptional regulator RhlR
Exopolysaccharides	QY43GL000985	(*algK*) Sel1-like repeat protein
	QY43GL001449	(*algA*) phosphomannose isomerase / guanosine 5'-diphospho-D-mannose pyrophosphorylase
	QY43GL001450	(*algF*) alginate o-acetyltransferase AlgF
	QY43GL001451	(*algJ*) alginate o-acetyltransferase AlgJ
	QY43GL001452	(*algI*) alginate o-acetyltransferase AlgI
	QY43GL001453	(*algL*) poly(beta-d-mannuronate) lyase precursor AlgL
	QY43GL001455	(*algX*) alginate biosynthesis protein AlgX
	QY43GL001456	(*algG*) outer membrane protein AlgE
	QY43GL001457	(*algE*) Alginate production outer membrane protein AlgE precursor
	QY43GL001458	(*algK*) alginate biosynthesis protein Alg44
	QY43GL001459	(*alg44*) alginate biosynthesis protein Alg8
	QY43GL001460	(*alg8*) alginate biosynthesis protein Alg8
	QY43GL001461	(*algD*) GDP-mannose 6-dehydrogenase AlgD
	QY43GL003958	(*algB*) two-component response regulator AlgB
	QY43GL004611	(*algU*) alginate biosynthesis protein AlgZ/FimS

Biological control mechanism	Gene Name	Protein Names
	QY43GL004879	(*algW*) AlgW protein
	QY43GL005552	(*algP/algR3*) alginate regulatory protein AlgP
	QY43GL005755	(*algQ*) Alginate regulatory protein AlgQ
	QY43GL005761	(*algR*) alginate biosynthesis regulatory protein AlgR
	QY43GL005762	(*algZ*) sigma factor AlgU
	QY43GL005823	(*algC*) phosphomannomutase AlgC
	QY43GL005993	(*algB*) two-component response regulator AlgB
antagonistic activities		
Salicylate	QY43GL000720	(*pchA*) salicylate biosynthesis isochorismate synthase PchA
	QY43GL000721	(*pchB*) salicylate biosynthesis protein PchB
Siderophores production		
Pyochelin	QY43GL000722	(*pchC*) pyochelin biosynthetic protein PchC
	QY43GL000723	(*pchD*) pyochelin biosynthesis protein PchD
	QY43GL000728	(*pchH*) putative ATP-binding component of ABC transporter
	QY43GL000729	(*pchI*) putative ATP-binding component of ABC transporter
	QY43GL000726	(*pchF*) pyochelin synthetase PchF
	QY43GL000727	(*pchG*) pyochelin biosynthetic protein PchG
	QY43GL000724	(*pchR*) transcriptional regulator PchR
Pyoverdine	QY43GL002741	(*pvdG*) PvdG
	QY43GL002763	(*pvdG*) putative thioesterase
	QY43GL002772	(*pvdI*) probable non-ribosomal peptide synthetase
	QY43GL002773	(*pvdJ*) PvdJ
	QY43GL002774	(*pvdD*) pyoverdine synthetase D
	QY43GL002777	(*pvdF*) pyoverdine synthetase F
	QY43GL002788	(*pvdA*) L-ornithine N5-oxygenase
Biofilm Formation	QY43GL001637	(*flgN*) putative export chaperone involved in flagellar synthesis
	QY43GL001638	(*flgM*) negative regulator of flagellin synthesis
	QY43GL001639	(*flgA*) flagellar basal body P-ring biosynthesis protein FlgA
	QY43GL004272	(*flgL*) flagellar hook-associated protein 3
	QY43GL004273	(*flgK*) flagellar hook-associated protein FlgK
	QY43GL004274	(*flgJ*) flagellar protein FlgJ
	QY43GL004275	(*flgI*) flagellar P-ring protein precursor FlgI
	QY43GL004276	(*flgH*) flagellar L-ring protein precursor FlgH
	QY43GL004277	(*flgG*) flagellar basal-body rod protein FlgG
	QY43GL004278	(*flgF*) flagellar basal-body rod protein FlgF
	QY43GL004279	(*flgE*) flagellar hook protein FlgE
	QY43GL004280	(*flgD*) flagellar basal-body rod modification protein FlgD
	QY43GL004281	(*flgC*) flagellar basal-body rod protein FlgC
	QY43GL004282	(*flgB*) flagellar basal body rod protein FlgB
(PGP) attributes		
Phosphate metabolism	QY43GL004163	(*phoQ*) hypothetical protein
	QY43GL004164	(*phoP*) transcriptional regulatory protein PhoP, regulator of virulence determinants

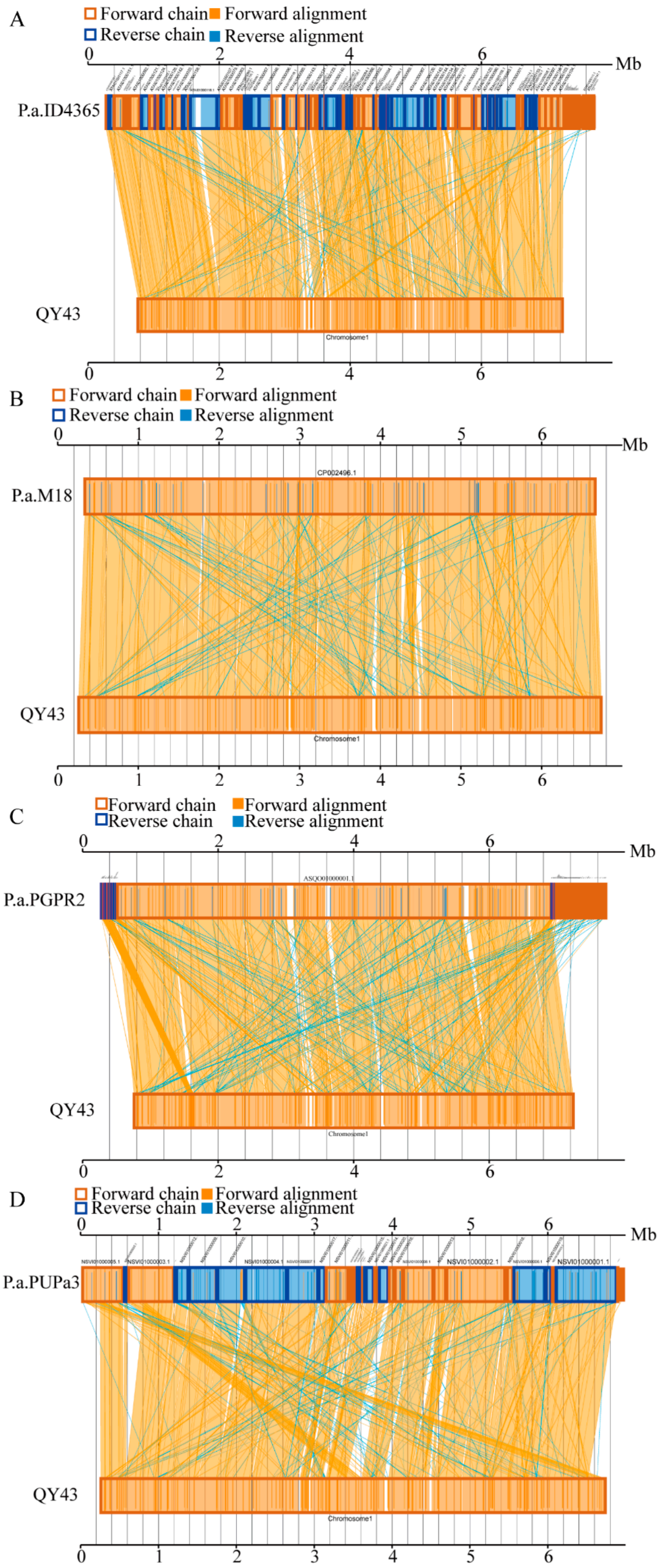

A
Forward chain
Forward alignment
Reverse chain
Reverse alignment
0
2
4
6
Mb
P.a.ID4365
QY43
Chromosome1
0
2
4
6
B
Forward chain
Forward alignment
Reverse chain
Reverse alignment
0
1
2
3
4
5
6
Mb
CP002496.1
P.a.M18
QY43
Chromosome1
0
1
2
3
4
5
6
C
Forward chain
Forward alignment
Reverse chain
Reverse alignment
0
2
4
6
Mb
ASQO01000001.1
P.a.PGPR2
QY43
Chromosome1
0
2
4
6
D
Forward chain
Forward alignment
Reverse chain
Reverse alignment
0
1
2
3
4
5
6
Mb
NSVI01000003.1
NSVI01000004.1
NSVI01000002.1
NSVI01000001.1
P.a.PUPa3
QY43
Chromosome1
0
1
2
3
4
5
6

Figure S3. Comparative genomics analysis of QY43 with ID4365, M18, PGPR2, and PUPa3. (A): QY43, ID4365 nucleic acid level synteny; (B): QY43, M18 nucleic acid level synteny; (C): QY43, PGPR2 nucleic acid level synteny; (D): QY43, PUPa3 nucleic acid level synteny. The lower sequence represents the tested genome, whereas the upper sequence represents the reference sequence genome. Yellow and blue boxes in both sequences indicate the positive and negative strands of the genome, respectively. Yellow and blue areas within the boxes represent the nucleotide sequences of this genomic region in the positive and negative strands, respectively. Yellow and blue lines in the middle region of the figure depict forward and reverse complement alignments, respectively.
